# A user satisfaction model for mobile government services: a literature review

**DOI:** 10.7717/peerj-cs.1074

**Published:** 2022-08-22

**Authors:** Abdulla Jaafar Desmal, Suraya Hamid, Mohd Khalit Othman, Ali Zolait

**Affiliations:** 1 Faculty of Computer Science and Information Technology, Universiti Malaya, Kuala Lumpur, Malaysia; 2 College of Information Technology, University of Bahrain, Sakheer, Bahrain

**Keywords:** Mobile government, Mobile government satisfaction, Online satisfaction, User satisfaction, Electronic satisfaction, Mobile service satisfaction, Electronic government, Electronic service

## Abstract

User satisfaction is essential for the success of an organisation. With the development of government service delivery through mobile platforms, a compatible measurement model must be found to measure user satisfaction with performing such services through a mobile government portal. Measuring user satisfaction with mobile government services is necessary nowadays due to the increasing popularity of smart devices. Research on mGovernment users’ satisfaction is lacking, leading to difficulties in understanding users’ expectations. In the present study, systematic literature reviews have been used to analyze users’ satisfaction with mGovernment portals and propose a comprehensive model compatible with such contexts. The results show that government agencies can evaluate users’ satisfaction using the proposed model of six quality constructs: usability, interaction, consistency, information, accessibility, and privacy and security. The study recommends improving the evaluation strategies of mGovernment portals regularly to ensure they fit with challenges. Measuring user satisfaction at mGovernment services encourages the user to perform the transactions through such online platforms, increasing the digitalization process and reducing the cost and efforts for both the service provider and end-users.

## Introduction

Customer satisfaction is a fundamental approach to quality management. Identifying the needs and expectations of different customer segments forms the basis for obtaining satisfied customers. Therefore, analysing customer satisfaction is a critical element in understanding the quality of organisations’ products and services. Consequently, these characteristics are adjusted to the quality demanded. Gundersen (2004) defined customer satisfaction as a post-consumption assessment decision taken by the customer related to a product or service. This evaluation process occurs based on the results of the “customer’s prepurchase expectation with the perception of performance during and after the consumption experience” (Oliver, 1980).

However, the development of information and communication technologies (ICT) has influenced how organisations deal with clients. Unlike traditional businesses, online organisations create virtual communication between service providers and clients through their websites or smart devices connected to the internet. Mobile government (mGovernment) service is a technology used nowadays by government agencies to deliver government services to the public by providing mobile application services. The technology of mGovernment is an extended form of e-Government to create an attractive and smart environment between the government service provider and the public (Chanana, Agrawal & Punia, 2016). Global government agencies transmit online services through mobile devices, but such services’ success or failure is based on users’ satisfaction. Online services are different from the offline environment in that they create different experiences (Verma, Chaurasia & Bhattacharyya, 2019), so applying offline customer satisfaction models to online platforms causes inaccurate results. Measuring the service quality of mGovernment is still at an early stage of investigation by academic researchers. Compatible service quality measurement scales that target the area of mGovernment are lacking, which causes difficulties in understanding the behaviours and expectations of users. Previous studies have discussed the concept of customer satisfaction with the electronic form (*e.g*., website retailing, electronic government services) by analysing the concept, identifying the constructs, and proposing models for each of the cases. The absence of analysing customer satisfaction in the field of mGovernment services leads government agencies to use other scales that are not compatible with this smart environment, which causes incorrect analysis and weak understanding of end-users.

This article aims to propose a measurement model for customer satisfaction with mGovernment services, that is guide the authors to review the theoretical base models of customer satisfaction that guide researchers for a clear view of the main elements associated with the concept. A review of the previous studies in online customer satisfaction has been conducted to enhance the current research by elaborating on customer satisfaction constructs in the online service environment. Since the targeted area is mGovernment services, the present study considers the uniqueness of the mobile services that guide the identification of the criteria to evaluate customer satisfaction with mGovernment service portals. The outcome of this research encourages online government agencies to understand user satisfaction and conduct regular updates of such services to meet users’ needs. Therefore, the main research question is, what is the appropriate measurement model that can be used to measure the customer satisfaction at mGovernment services?

### Literature review

#### The concept of offline customer satisfaction

The definitions of the satisfaction concept described by previous literature from different approaches are based on cognitive and practical aspects that indicate the transaction’s specific character. Varying definitions in the research scope of usability create difficulties for researchers to analyse the usability concept’s origin, develop measurement scales, or critique empirical results (Giese & Cote, 2000). The disconfirmation theory is considered to deal with usability confusion as it is a proper technique for the simplicity of operationalisation. This approach located in the cognitive perspective implies that satisfaction results from comparing performance and related standards (Oliver, 1997). Understanding customer satisfaction is associated with marketing studies and practice in Cardozo’s (1965) research on customers’ efforts, expectations, and satisfaction. Since that time, many attempts have been made to explain and measure customer satisfaction, but to date, no standard definition of the concept has been agreed upon among researchers (Park et al., 2019; Dianat et al., 2019; Sandro et al., 2019). One of the purposes of customer satisfaction that Gundersen, Heide & Olsson (1996, 74) stated is a “post-consumption evaluative judgment concerning a specific product or service.”

Achieving customer satisfaction can benefit organisations, for example when customers return to buy the product or service again. Therefore, the organisation obtains customer loyalty and creates the possibility to deal with a service provider in the future (Caggiani et al., 2018). When a customer is satisfied with an organisation, the positive experience is shared with the customer’s relatives and friends. The satisfied customer leaves the positive values that obtains a particular place as a profit in the market (Tzavlopoulos et al., 2019).

Customer satisfaction is constructed on three main elements: (i) perceived performance, (ii) expectations, and (iii) satisfaction level. The first element, perceived performance, refers to the performance in terms of value delivery that the client obtains after acquiring a product or service. In other words, it is the result that the customer perceives from the product or service. The second element is expectations, which are the hopes that customers have due to one or more situations, such as promises made by organisations, previous experiences, opinions of others, and other competitors’ guarantees. The third element is the level of satisfaction that is experienced after purchasing the product or service (Hu, Kandampully & Juwaheer, 2009).

Although customer satisfaction is a metric that helps organisations to enable their products or services to meet or exceed consumer expectations (Othman, Hamzah & Abu Hassan, 2020), all of the values must be necessary for customer satisfaction and recognise how that helps to manage and improve the business (Mannan et al., 2019; Tzavlopoulos et al., 2019).

#### Electronic customer satisfaction

The criteria for defining the concept of electronic customer satisfaction through online platforms are grounded in traditional business. One of the definitions of e-customer satisfaction is “the contentment of the customer concerning his or her prior purchasing experience with a given electronic commerce firm” (Anderson & Srinivasan, 2003, 125). A study by Novak, Hoffman & Yung (2003) defined the concept of e-customer satisfaction as a “cognitive state experienced during navigation”, (p.22) while other studies define it as a psychological state that is constructed based on online interaction through the website (Rose et al., 2012, 309). Trevinal & Stenger (2014) described e-customer satisfaction from a shopping practice viewpoint and defined it as the interaction process that results between customers, shopping practice tools, and online portals. As mentioned in the definitions, electronic customer satisfaction is constructed based on the emotional aspect of the user’s interaction with online portals.

However, previous research proposed measuring scales for electronic customer satisfaction by identifying the scale dimensions associated with online website features. Standard dimensions include “ease of use through these scales,” which describes a customer’s ability to perform the online transaction with few difficulties. The dimension “ease of use” uses the exact name of the proposed measurement scale in studies by Reynolds (2011), Tang & Wang (2004), and Cho & Park (2001). In other cases, “ease of use” reflects the status of the system, such as the study by Liu & Arnett (2000) that uses the dimension of “ease of the system.” A study by Novak, Hoffman & Yung (2003) uses the dimensions of “ease of contact, easy ordering, easy of cancellation,” all of which return to the practice of “ease of use.” Measuring the quality of information is common: James & Sammy (1983) label it “information product,” Reynolds (2011) labels it “format,” Chen (2002) labels it “informativeness,” and Novak, Hoffman & Yung (2003) label it “information quality.” The importance of customer support in an online environment is considered on most e-customer satisfaction scales; for example, James & Sammy (1983) use the dimension name “vendor support”, Novak, Hoffman & Yung (2003) use the dimension name “ease of contact”, Tang & Wang (2004) use the dimension name “customer support”, Cho & Park (2001) use the dimension name “customer service,” and Parasuraman, Zeithaml & Malhotra (2005) use the dimension name “contact.” The overall website design influences e-customer satisfaction, as used in the measurement scales of Reynolds (2011), Liu & Arnett (2000), Cho & Park (2001), and Evanschitzky et al. (2004).

However, based on previous literature on electronic customer satisfaction, these proposed scales described the general virtual environment that may influence the level of customer satisfaction.

#### MGovernment services

MGovernment is a form of government service delivered through smart device applications (apps) and uses interactive SMS services to reach the public flexibly and comfortably. MGovernment is at the initial stages of delivering mobile applications services, and many countries have updated their regulation policies to be compatible with such services. The responsibilities of mGovernment services are not separate from e-Government services. Both e-Government and mGovernment portals aim to provide government services to the public, such as health services, education services, employee services, and business services. Studies that consider mGovernment as an extension of e-Government include Kassen (2017) and Santa, MacDonald & Ferrer (2019), while other scholars regard it as a “separate channel” that provides the services through smart wireless (Janita & Miranda, 2018; Chen, Vogel & Wang, 2016). MGovernment service is a more flexible way to deliver government services to the public due to the low cost of smart devices, hand-held devices, and ease of use for most people. The success of services on mGovernment portals depends on user satisfaction because users are the central element in the online services environment. Various quality dimensions are associated with the evaluation of mGovernment service quality, and user satisfaction is one of them. The quality dimension of user satisfaction is required to construct a broad evaluation scale that can measure the smart devices’ nature with consideration of unique features such as portability, personalisation, limitation of technical features, input features, location, and interaction features (Demir et al., 2020; Khan, Zubair & Malik, 2019). The reliability is one of the mGovernment quality factor that has the ability to measure the system performance using the attributes of “timeliness, accuracy, error-free, service promise, and confidentiality” (Desmal et al., 2019a, 2019b, 2019c). Using other online measurement scales such as e-Government, e-Commerce, and eRetailing in mGovernment services can lead to difficulties in understanding user satisfaction because each of the contexts has its features and requires a particular measurement scale (Song & Christen, 2019). Jaafar Mohamed et al. (2019) stated that the mGovernment is an electronic interaction portal that can communicate between the government service provider and the user, where the quality can be measured using the quality factors of “user control, synchronicity, two-way communication, and responsiveness”. Desmal, Othman & Hamid (2021) formulate the uniqness of the mGovernment portal among the factors of “location-based services, smart interactions, consistency, accessibility, and efficiency”.

### Proposed model for customer satisfaction with mGovernment

The present study aims to understand user satisfaction by proposing a compatible measurement scale model for the portal of mGovernment services. Due to the lack of studies directly reporting on the field of user satisfaction with mGovernment (Al-Hubaishi, Ahmad & Hussain, 2017; Shareef et al., 2014), the literature from other near areas such as e-Government and e-Commerce was reviewed to construct a model for measuring user satisfaction with mGovernment portals. These portals are unique and require more attention to ensure continued use by users. To achieve user satisfaction, it is essential to conduct a regular review of the service delivery process to users, which helps government agencies reengineer the strategy to meet users’ expectations.

Considering the unique features of mGovernment portals, a study by Al-Hubaishi, Ahmad & Hussain (2017) proposed a measurement scale to measure the quality of services and uses the dimensions of “interaction quality, environment quality, information quality, system quality, network quality, and outcome quality”. Shareef et al. (2014) proposed a model for service quality by mGovernment, consisting of four dimensions: “connectivity, interactivity, understandability, and authenticity”. In the field of mobile banking, Karjaluoto et al. (2018) measured end mobile application user satisfaction by using the dimensions of “personal innovativeness, self-congruence, perceived risk, new product novelty, perceived value, overall satisfaction, commitment”, while Khan, Lima & Mahmud (2018) use the dimensions of “tangibility, reliability, responsiveness, assurance, and empathy” to measure customer satisfaction in the field of mobile application banking. In other sectors, a study by Othman & Razak (2010) aimed to measure the mobile application satisfaction of school dental service by using the dimensions of “technical competency, interaction, efficiency, environment.” Therefore, to measure the satisfaction of mGovernment users, the present article proposes an mGovernment satisfaction scale consisting of the unique dimensions relevant to characteristics of service-based smart devices.

#### Usability

The term usability refers to “the extent to which specified users can use a product to achieve specified goals with effectiveness, efficiency, and satisfaction in a specified context of use” (International Organization for Standardization, 2013). When the software or mobile applications are released to the markets, organisations are expected to accept users. That degree depends on characteristics that are considered essential by each user. Dynes & Whisler (2006) defined the concept of ease of use as the “degree to which users can use the system which the skills, knowledge, stereotype, and experience they can bring to bear”. However, in previous research, Liu & Arnett (2000), Novak, Hoffman & Yung (2003), Tang & Wang (2004) and Reynolds (2011) used usability/ease of use to measure electronic customer satisfaction. Government agencies use mobile applications to deliver services to members of the public who have different educational levels. Considering mGovernment portals’ usability features, maximum users perform transactions through such services due to their satisfaction. MGovernment portals have unique technical features to measure usability that are different from other electronic services (Jaafar Desmal, 2017; Desmal et al., 2019a; Belgaum et al., 2021). Based on previous theories, the following hypothesis is proposed:

H1: Usability has a significant impact on users’ satisfaction with mGovernment services.

#### Interaction

Interaction refers to measuring user satisfaction while interacting with the service and system through the mGovernment portal during the delivery of such services. Kim et al. (2015) argue that the mobile application is considered as the main channel that conducting interactivity between the service provider and end-users. It means that mobile interactivity is requested from user to machine to perform a service (Lee, Lee & Kim, 2019; Monica et al., 2022; Isidro & Ashour, 2022). The interaction element is intangible and occurs during service delivery to the customer, which influences customer satisfaction. Since goods are not considered in mGovernment portals, the government agency’s service provider aims to satisfy users by targeting their expectations, affecting the relationship between them (Lu, Wu & Hsiao, 2019; Ashour, Hussin & Mahar, 2008). Measuring the interactivity dimension cannot be done in isolation; it is a complex dimension consisting of related elements, processes, operations, and perceptions. In this context, Heeter (1989) uses six elements to construct a complete context of interactivity, which are “complexity of choice available, user’s effort, user’s responsiveness, monitoring of information use, ease of adding information, and facilitation of interpersonal communication”. To meet users’ satisfaction with mGovernment, managers must pay more attention to their employees’ skills to provide the best service level to the public. Interaction with the mGovernment portal can be through online chat, voice, video, or email (Yang & Zeng, 2018; Cupertino et al., 2019; Senthil Kumar et al., 2022; Uma Maheswari, Aluvalu & Mudrakola, 2022). Providing more options for interactions with mGovernment means the users can get in touch quickly with a government agency, which influences the continued use of such services.

H2: Interaction has a significant impact on users’ satisfaction with mGovernment services.

#### Consistency

The concept of consistency in mobile application service refers to the compatibility of application elements, design, interface, navigation, and operational process with the nature of services (Li et al., 2019). Measuring the consistency of service applications is necessary due to smart devices’ unique features that may create difficulties for end-users. Heinrich et al. (2018) argue that any desktop or application software’s goal is to keep end-users satisfied with such services and avoid relearning the transaction process in the future. Introducing mobile service features that are not familiar due to lack of consistency can impact users’ efforts when using a mobile device (Vidyasankar, 2018; Alansari et al., 2020; Shuja, Humayun & Rehman, 2021). Previous researchers have measured the dimension of consistency to measure the satisfaction of online users. A study by Reynolds (2011) uses two main dimensions (content and format) to measure the concept of consistency in the field of electronic commerce websites and its impact on user satisfaction Chen (2002) uses the dimension of “organization”. Liu & Arnett (2000) use the dimension of “quality of system design” to measure consistency. This shows that the concept of consistency is vital when measuring online user satisfaction. The introduction of parallel steps of service performance lets users understand the overview of functional process that assists in preparing any documents or information requirements before starting the service (Lai & Liu, 2019). Consistency can help improve mobile services’ usability because they can deduce the application if it looks like other application structures (Li et al., 2019; Shuja et al., 2021b). Consistency in mobile services must be practised in the general design to avoid all kinds of failures during the execution of the application (Jung, 2017; Shuja et al., 2021a). Inconsistent design of a mobile application service may create a messy application that will be disappointing for its users (Park et al., 2019; Jung, 2017). Ensuring consistency of mGovernment service allows each section of the application to develop perfectly and generate exceptionally fluid flow. Based on previous studies, the following hypothesis is proposed:

H3: Consistency has a significant impact on users’ satisfaction with mGovernment services.

#### Information

The term information has been defined from the quality field view as “intrinsic, contextual, representational, and accessibility” (Lee et al., 2002), which influences interconnected elements such as the format, currency, and completeness of the information. Some researchers measure the satisfaction of users with online platforms by using alternative dimensions. For example, James & Sammy (1983) use the term “service information product”, Reynolds (2011) uses the term “content”, and Cho & Park (2001) use the term “product information”. When services are delivered through smart devices, many attributes affect the satisfaction of users, and the dimension of information is one of the main elements (Riesener et al., 2019; Heinrich et al., 2018; Gharib & Giorgini, 2019; Sayed & Ashour, 2022). The information provided to users through the service provider’s online platform must enhance their understanding, be received on time and be accurate and understandable (Oliveira & Chan, 2019; Torres & Sidorova, 2019; Gharib & Giorgini, 2019). In mGovernment portals, the information may depend on multiple government agencies to process the transaction before sending the final transaction to end-users, which requires quick processing, accuracy, and complete processing of the service to ensure users’ satisfaction. Based on previous studies, the following hypothesis is proposed:

H4: Information has a significant impact on users’ satisfaction with mGovernment services.

#### Accessibility

The concept of accessibility refers to the use of online services, products, frameworks, or resources in an effective, efficient, and satisfying way by people with different abilities (Işeri, Uyar & Ilhan, 2017; Yoon et al., 2016; Ashour et al., 2014; Alansari, Siddique & Ashour, 2022). The concept of ICT accessibility is essential to ensure equal opportunities for all people to use and access online resources, products, and services (Crespo, Espada & Burgos, 2016). Previous research shows that websites do not meet the needs of people with various disabilities (Southwell & Slater, 2012; Lewis, 2013; Lazar, Olalere & Wentz, 2012), which causes difficulties for these people to utilise online services. A statistic revealed that 36 million people are blind (Cupertino et al., 2019). Considering this figure, creating standards to offer accessibility options for government services, especially mGovernment services, will enhance most people’s ability to perform the transactions using their smart devices and save time and effort. The authors of the present study noted a lack of measuring the term of accessibility in mGovernment portals. Hence, the current study measures accessibility in how the application of mGovernment service portals provides options to access and perform the services with less time and effort by users. Based on these findings, the following hypothesis is proposed:

H5: Accessibility has a significant impact on users’ satisfaction with mGovernment services.

#### *Privacy and* security

The online environment's two main elements are privacy and security (Widjaja et al., 2019; Barth et al., 2019). These two elements are essential to satisfy end-users (Abedi, Zeleznikow & Brien, 2019). Information privacy is understood as the control exercised by the user over their information to prevent unauthorised parties from accessing it (Cui et al., 2019). This information may include data, photos, and files (Merhi, Hone & Tarhini, 2019). Simultaneously, the term information security refers to preventing all threats that affect online transactions (Alomar, Alsaleh & Alarifi, 2019). Ensuring complete privacy and security are essential aspects to be considered by users and service providers for transaction processing (Liao & Shi, 2017). When privacy or security in online services is weak, the users will not conduct any type of transactions, especially when related to financial data (Ma, Chen & Zhang, 2019; Sá et al., 2017; Widjaja et al., 2019). Users’ satisfaction with online services is affected by the power of privacy and security (Cui et al., 2019; Barth et al., 2019). Since mGovernment is provided through mobile devices, it is necessary to ensure that the mobile application is developed with highly professional techniques to ensure public satisfaction with mobile services. Based on these findings, the following hypothesis is proposed:

H6: Privacy and security have a significant impact on users’ satisfaction with mGovernment services.

**Figure 1:**
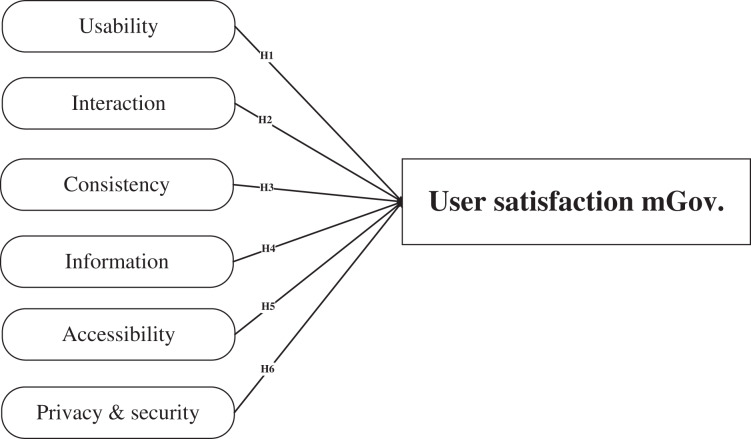
Proposed model to measure users’ satisfaction with mGovernment services.

The output from the previous literatures guide the authors to formulate the proposed model to measure user’s satisfaction with mGovernment services as shown at Fig. 1.

## Methodology

To propose a model that can measure user satisfaction with mGovernment service portals, a review of the literature was conducted to obtain a comprehensive view of the present approaches and identify the study’s requirements. The selected articles are from the year 2010 or later and measured or evaluated user satisfaction with various online platforms. The reason behind the selected starting period is that the majority of the studies at the field of online services were started analysing the importance of measuring the satisfaction of online users. The first step was the collection of literature from the digital libraries of Emerald, SAGA, ScienceDirect, Scopus, Taylor and Francis, and Web of Science. The top search strings used in the present research were “mobile government services”, “mobile application satisfaction”, “mobile satisfaction”, “application satisfaction”, “e-satisfaction”, “electronic satisfaction”, “website satisfaction”, “e-service satisfaction”, “electronic service satisfaction”, “online satisfaction”, “e-government satisfaction”, “electronic government satisfaction”, “user satisfaction”, and “customer satisfaction”. The selection criteria of the articles guides the authors to formulate and validate the proposed satisfaction model for mGovernment services that consists of the basic elements of measuring the satisfaction of online users. Most of the publications in the literature reviews appeared between 2015 and 2019. Due to the absence of literature in the field of satisfaction with mGovernment portals, the present study used fields near to mGovernment to guide the authors in proposing the targeted model. The type of publications used were short articles and full articles from peer-reviewed sources.

The abstracts were reviewed based on the criteria below:

1. The research focuses on online services (desktops or mobile devices).
2. The research aims to measure or propose a model or framework for user or customer satisfaction.
3. The research evaluates user or customer satisfaction with leads to propose the constructs, sub-dimensions or items for a model or framework.

The publications that did not meet the above three criteria were removed from the current research analysis. Publications were excluded according to the criteria below:

1. The research measures customer satisfaction based on an offline service environment.
2. The research does not consider satisfaction as a primary model or sub-dimension.
3. The research does not propose a unique model or framework to measure user or customer satisfaction.

**Figure 2:**
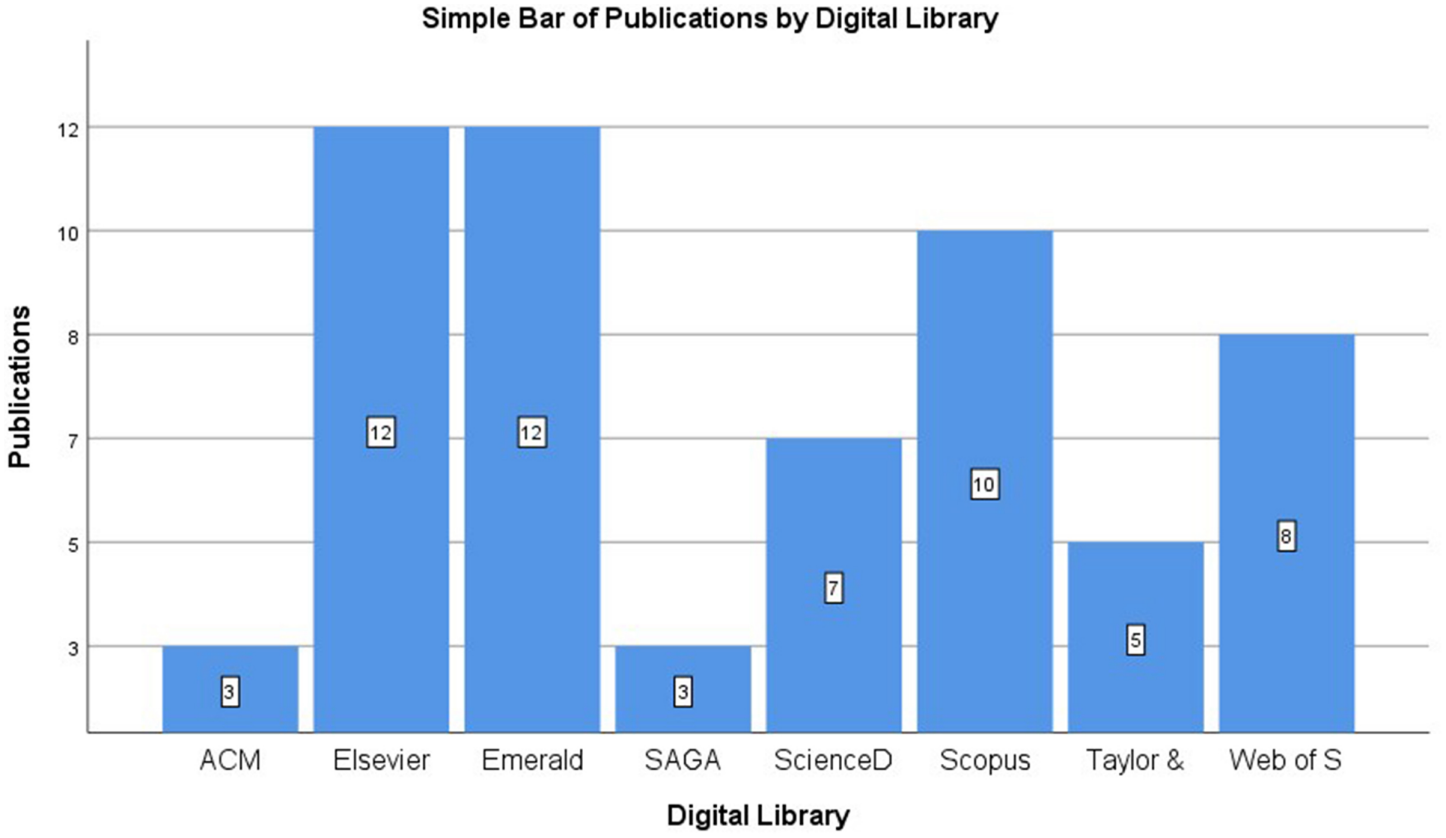
Number of publications per digital library.

In conducting the literature review, the abstract for each article was reviewed to ensure the scope of the research fit with the area of the present research. Table 1 and Fig. 2 show the number of publications in the literature review.

**Table 1:** Number of publications per digital library.

Digital library	2015	2016	2017	2018	2019	Total
ACM	0	1	0	1	1	3
Emerald	0	2	1	4	5	12
SAGA	1	0	1	0	1	3
ScienceDirect	1	1	0	2	3	7
Scopus	0	2	2	5	1	10
Taylor and Francis	1	0	3	1	0	5
Web of Science	1	1	1	1	4	8
Elsevier	2	0	1	3	6	12
Total	6	7	9	17	21	60

**Figure 3:**
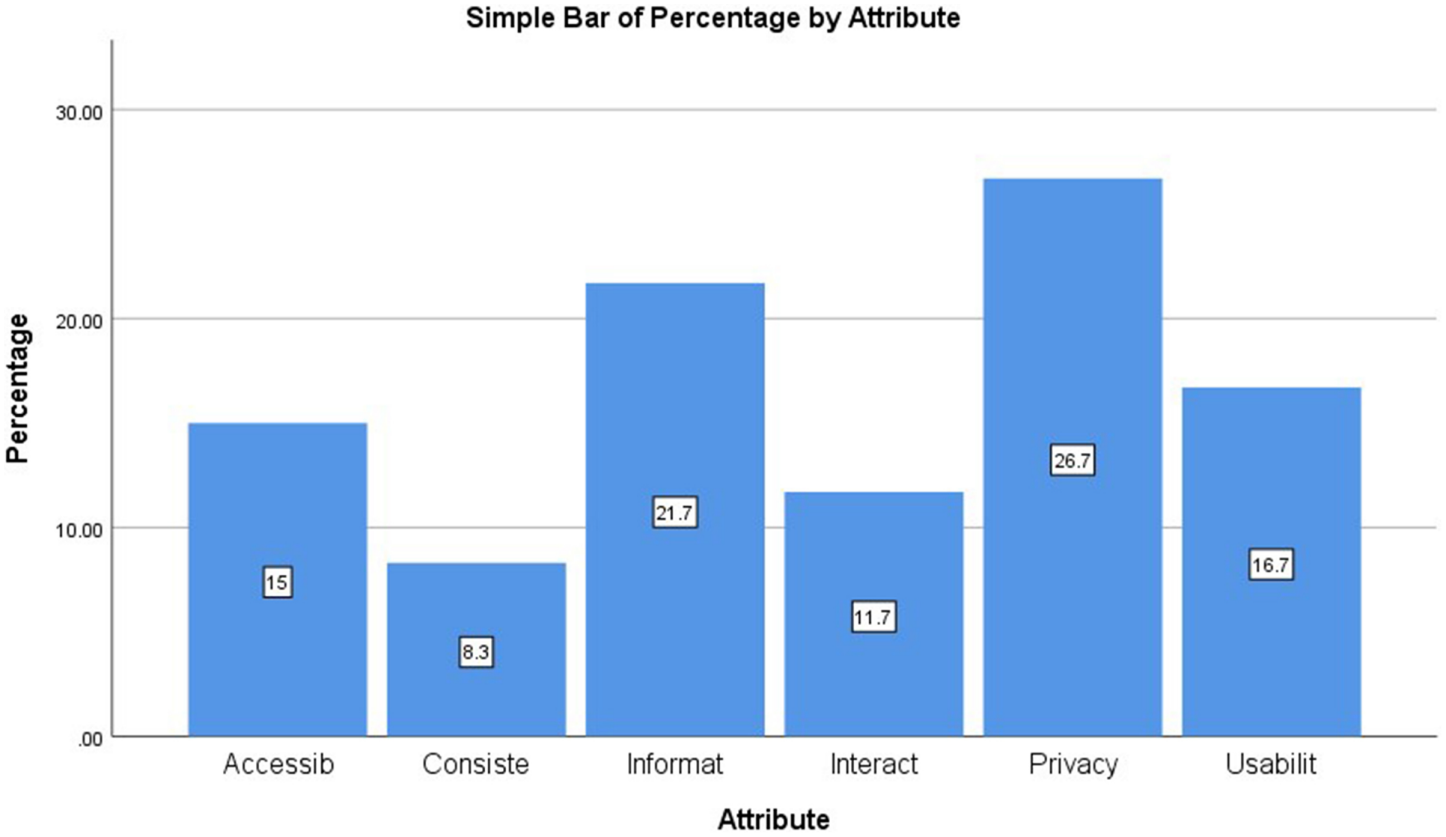
Percentage of extracted attributes from previous literature related to online user satisfaction.

The attributes of the user satisfaction model at mGovernment service are extracted from previous related literature. As shown in Table 2 and Fig. 3, the attribute of “consistency” has little literature when its analyses are based on the mobile application, while the attribute of “privacy and security” has the most significant percentage of focused literature.

**Table 2:** Total and percentage of extracted attributes from previous literature related to online user satisfaction.

Attribute	% of studies	Number of studies
Usability	16.7	10
Interaction	11.7	7
Consistency	8.3	5
Information	21.7	13
Accessibility	15.0	9
Privacy and security	26.7	16
Total	100.0	60

**Figure 4:**
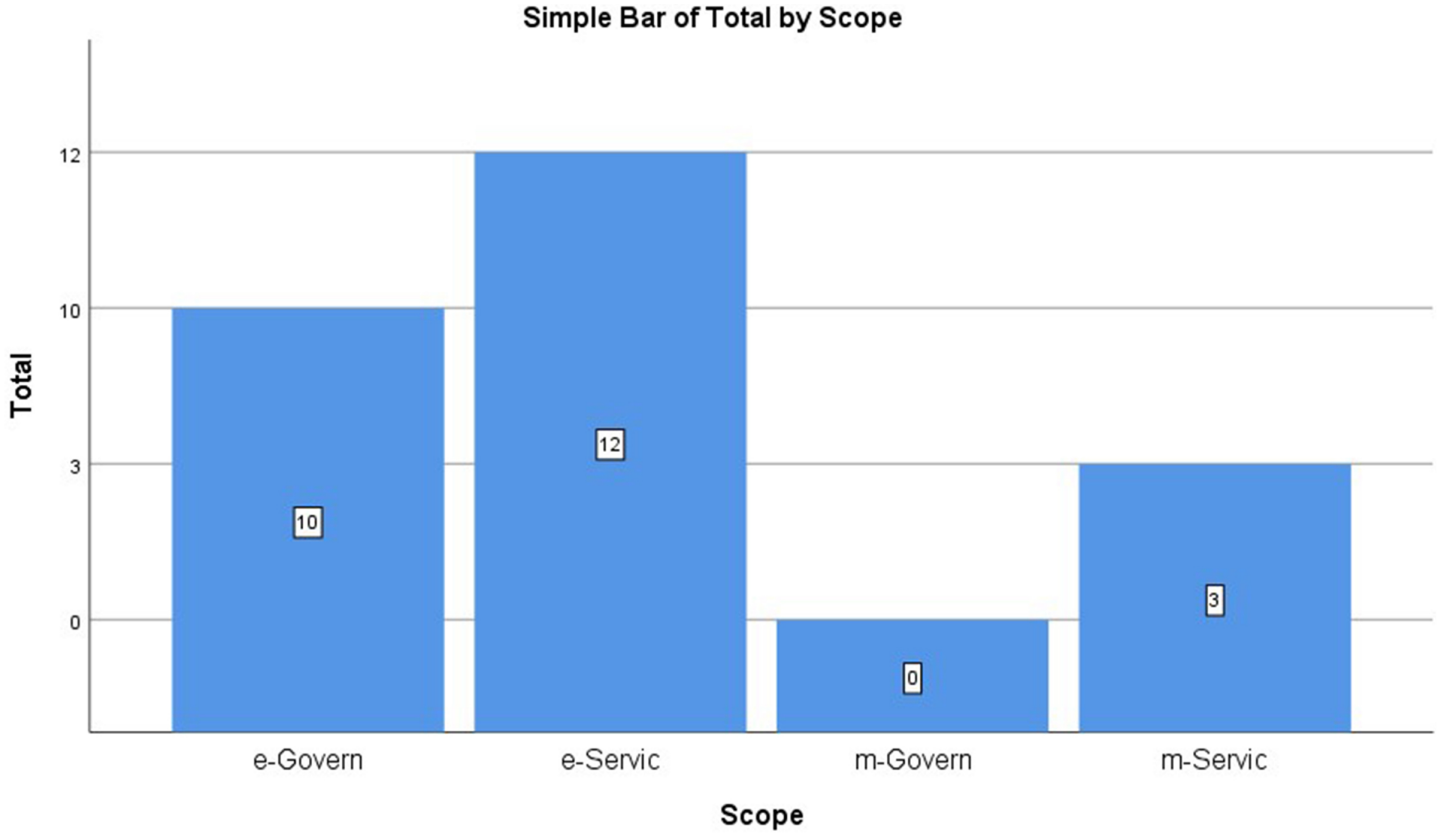
User satisfaction models proposed by literature per scope.

Models of online services from previous studies have been classified according to the type of user platform (desktop/online and mobile). However, as shown in Table 3 and Fig. 4, the highest number of available proposed models belong to the category of e-Services with a percentage of 48%, while no model measures user satisfaction with mGovernment service platforms. The reason for writing the present article is to study, analyse and propose this with its attributes.

**Table 3:** User satisfaction models proposed by literature per scope.

Scope	%	Total studies
e-Service	48.0	12
e-Government	40.0	10
m-Service	12.0	3
mGovernment	0.0	0
Total	100.0	25

### Research implications

The proposed model of user satisfaction on mGovernment service platforms can be used for further study of the service delivery process from the perspective of end-users (see Appendix Table 3). Using other online models in the environment of mGovernment leads to difficulties in understanding exact levels of user satisfaction, which affects the continued use and the future of applying such services on mGovernment portals. The present article provides the model proposed to be used in the context of mGovernment services while considering the unique features of this type of service. A total of six attributes of the proposed model were described to enhance the government agencies' measure of each aspect that may influence the level of end-user satisfaction (see Fig. 1). Researchers and practitioners can use this model for further research in terms of quantitative or qualitative studies to analyse the attributes that influence the satisfaction of end-users with mGovernment portals to help government agencies focus on these most important attributes affecting the process of service delivery.

### Research limitations

The present research consists of six constructs related to the model of user satisfaction. These constructs are extracted based on theories from previous literature reviews, and further practical studies are required to measure the impact of each construct on user satisfaction.

## Conclusions

User satisfaction is a source of success in every sector. MGovernment services aim to deliver government services through smart devices to the public and to ensure that the end-users are satisfied with such services. It is essential to provide and reengineer the process of service delivery until the public is satisfied. Previous literature shows a lack of focus on mGovernment services in terms of user satisfaction. When it comes to measuring the delivery of services based on mobile devices, it is crucial to find the attributes that fit the unique features of mobile devices, such as portability, small screens, limited features compared with desktop devices, wireless access, and touch screens. Hence, using other models to measure user satisfaction with mGovernment can lead to more difficulties and inaccurate results. Each model has its features and attributes and is constructed based on its context. In this case, based on previous literature reviews, the current article proposed a comprehensive model with a total of six related attributes (usability, interaction, consistency, information, accessibility, and privacy and security) that can measure user satisfaction with mGovernment. It is a guide for decision-makers at government agencies to improve the services on mGovernment portals based on user satisfaction.

## Supplemental Information

10.7717/peerj-cs.1074/supp-1 Supplemental Information 1 Measurement scales of e-Customer Satisfaction.

10.7717/peerj-cs.1074/supp-2 Supplemental Information 2 Measurement scales of m-User Satisfaction.

10.7717/peerj-cs.1074/supp-3 Supplemental Information 3 Proposed measurement scales of m-User Satisfaction with mGovernment services.

## Additional Information and Declarations

### Data Availability

The following information was supplied regarding data availability: The raw data is available in the Supplemental Files.
