# Supplementary material for: A user satisfaction model for mobile government services: a literature review
Source: PeerJ Comput Sci. 2022 Aug 22;8:e1074. doi: 10.7717/peerj-cs.1074 (PMC9455267; doi:10.7717/peerj-cs.1074)
Supplement: Supplemental Information 1 [file peerj-cs-08-1074-s001.docx]

| **#** | **Scale** | **Author** | **Context** | **Main dimensions** | **Total constructs** |
| --- | --- | --- | --- | --- | --- |
| 1 | UIS – User Information Satisfaction | (James and Sammy 1983) | Electronic Commerce | Electronic data processing staff, service information product, vendor support, information product, involvement | 156 |
| 2 | EUCS – End-User Computing Satisfaction | (Reynolds 2011) | Electronic Commerce | Content, accuracy, format, ease of use, timeline | 12 |
| 3 | ATS – Attitude Toward Site | (Q. Chen 2002) | General Website | Entertainment, informativeness, organisation | 6 |
| 4 | Factors Associated with Website Success | (Liu and Arnett 2000) | Electronic Commerce | Quality of information & service, use of the system, playfulness, quality of system design | - |
| 5 | Compelling online experiences | (Novak, Hoffman, and Yung 2003) | Online retailing | Ease of contact, easy ordering, payment returns, easy of cancellation, customer support, cutting-edge, variety, information quality, reliability, security, low price | - |
| 6 | CIS – Customer Information Satisfaction | (Tang and Wang 2004) | General Website | Customer support, security, ease of use, digital products/services, transactions and payment, information content, innovation | 21 |
| 7 | ECUSI – Electronic Commerce User‐Consumer Satisfaction Index | (Cho and Park 2001) | Electronic Commerce | Product information, consumer service, purchase result and delivery, site design, purchase process, product merchandising, delivery time and change, payment method, ease of use | 51 |
| 8 | WCS – Web Customer Satisfaction | (McKinney, Yoon, and Zahedi 2002) | Online retailing | Understandability, reliability, usefulness, access, usability, navigation | 16 |
| 9 | WUS – Website User Satisfaction | (Muylle, Moenaert, and Despontin 2004) | Electronic Commerce | Information relevancy, information accuracy | 34 |
| 10 | Online shopping & financial service sites | (Evanschitzky et al. 2004) | Internet retail & financial services | Convenience, merchandising – product offering, merchandising – product information, design, financial security, customer satisfaction | 19 |
| 11 | E-S-QUAL | (Parasuraman, Zeithaml, and Malhotra 2005) | e-Service Quality | Efficiency, system availability, fulfilment, privacy | 22 |
| 12 | E-RecS-QUAL | (Parasuraman, Zeithaml, and Malhotra 2005) | e-Service Quality | Responsiveness, compensation, contact | 11 |
