# Supplementary material for: A user satisfaction model for mobile government services: a literature review
Source: PeerJ Comput Sci. 2022 Aug 22;8:e1074. doi: 10.7717/peerj-cs.1074 (PMC9455267; doi:10.7717/peerj-cs.1074)
Supplement: Supplemental Information 2 [file peerj-cs-08-1074-s002.docx]

| **#** | **Scale** | **Author** | **Context** | **Main dimensions** | **Total constructs** |
| --- | --- | --- | --- | --- | --- |
| 1 | Satisfaction with school dental service by Mobile Dental Squads | (N. Othman and Razak 2010) | School Dental Service | Technical competency, interaction, efficiency, environment | 22 |
| 2 | SQ & CS of Mobile Banking in Bangladesh | (A. G. Khan, Lima, and Mahmud 2018) | Mobile Banking | Tangibility, reliability, responsiveness, assurance, and empathy | 14 |
| 3 | Mobile financial services apps | (Karjaluoto et al. 2018) | Mobile Banking | Personal innovativeness, self-congruence, perceived risk, new product novelty, perceived value, overall satisfaction, commitment | 30 |
