# Supplementary material for: A user satisfaction model for mobile government services: a literature review
Source: PeerJ Comput Sci. 2022 Aug 22;8:e1074. doi: 10.7717/peerj-cs.1074 (PMC9455267; doi:10.7717/peerj-cs.1074)
Supplement: Supplemental Information 3 [file peerj-cs-08-1074-s003.docx]

| **Dimensions** | **Supporting studies** | **Findings of previous studies** |
| --- | --- | --- |
| Usability | (Reynolds 2011),(Liu and Arnett 2000), (Novak, Hoffman, and Yung 2003), (Tang and Wang 2004), (Cho and Park 2001), (McKinney, Yoon, and Zahedi 2002) | It has been found that usability is influencing the level of customer satisfaction at electronic portals.  A common recommendation among previous studies is to construct the environment in the form of an electronic portal with less effort and fewer difficulties to be flexible and easy to use by customers. |
| Interaction | (Kim et al. 2015),(S. H. Lee, Lee, and Kim 2019), (Lu, Wu, and Hsiao 2019),(Yang and Zeng 2018), (Cupertino et al. 2019) | The main finding of previous studies shows that the concept of interaction at services based mobile enhanced the relationship between the service provider and end-users, which influences users' satisfaction and encourages them for continued use. |
| Consistency | (Chunlin et al. 2019),(Heinrich et al. 2018),(Vidyasankar 2018), (Lai and Liu 2019), (Park et al. 2019) | The consistency of mobile applications is enhanced by the application design, interface, navigation, and operational process of mobile application services.  In terms of users’ satisfaction, previous studies recommended ensuring mobile application consistency that influences the continued use of users. |
| Information | (Heinrich et al. 2018), (Gharib and Giorgini 2019), (Riesener et al. 2019), (Oliveira and Chan 2019) | It finds that information at online services is considered an essential element that affects the users’ satisfaction. Such criteria related to information are the format of information at online service platforms, currency, and information completeness. |
| Accessibility | (Yoon et al. 2016), (Işeri, Uyar, and Ilhan 2017), (Crespo, Espada, and Burgos 2016), (Cupertino et al. 2019) | The concept of accessibility refers to online services, products, frameworks, or resources in an effective, efficient, and satisfying way by people with different abilities. |
| Privacy and security | (Barth et al. 2019), (Widjaja et al. 2019), (Cui et al. 2019), (Ma, Chen, and Zhang 2019) | Privacy and security are essential aspects to be considered by users and service providers to ensure complete privacy and security during transaction processing.  Since mGovernment is provided through mobile devices, it is necessary to ensure that the mobile application is developed with high professional techniques to ensure public service satisfaction. |
